# Concordance and Clinical Outcomes Improvement Following Oral Hygiene Motivation: A Systematic Review and Report of the Workshop of the Italian Societies of Dental Hygiene

**DOI:** 10.1155/2024/8592336

**Published:** 2024-10-16

**Authors:** Antonia Abbinante, Anna Antonacci, Michela Antonioni, Andrea Butera, Matteo Castaldi, Silvia Cotellessa, Caterina Di Marco, Martina Gangale, Rossana Izzetti, Maurizio Luperini, Carolina Maiorani, Gianna Maria Nardi, Alice Ravoni, Silvia Sabatini, Sandro Sestito, Augusta Virno, Filippo Graziani

**Affiliations:** ^1^Italian Association of Dental Hygienists (AIDI), Complex Operative Unit of Stomatology, Department of Interdisciplinary Medicine, University of Bari “Aldo Moro”, Bari, Italy; ^2^Department of Clinical and Experimental Medicine, University of Foggia, Foggia, Italy; ^3^Italian Association of Dental Hygienists (AIDI), Italy; ^4^Academy of Advanced Technologies in Oral Hygiene Sciences (ATASIO), Department of Oral and Maxillofacial Sciences, Sapienza University of Rome, Rome, Italy; ^5^Unit of Dental Hygiene, Section of Dentistry, Department of Clinical, Surgical Diagnostic and Pediatric Sciences, University of Pavia, Pavia, Italy; ^6^Academy of Advanced Technologies in Oral Hygiene Sciences (ATASIO), Italy; ^7^Italian Society of Oral Hygiene Sciences (SISIO), Italy; ^8^National Register Commission of Dental Hygienists, Italy; ^9^Department of Medicine and Technological Innovation, University of Insubria, Varese, Italy; ^10^Department of Surgical, Medical and Molecular Pathology and Critical Care Medicine, University of Pisa, via Savi 10, Pisa 56126, Italy; ^11^Department of Surgical, Medical, Dental and Morphological Sciences, University of Modena and Reggio Emilia, Modena, Italy; ^12^Private Practice, Italy; ^13^Department of Surgical, Medical, Dental and Morphological Sciences, University of Modena and Reggio Emilia, Modena, Italy; ^14^ATS, U.N.I.D., Udine, Italy

**Keywords:** motivation, oral hygiene, periodontal diseases, systematic review, treatment adherence and compliance

## Abstract

**Aim:** A workshop on concordance and oral hygiene was held in February 2024. To address the topic, a systematic review aimed at investigating the effectiveness of motivational interventions in improving oral hygiene and focusing on periodontal clinical indices outcomes was designed.

**Materials and Methods:** A comprehensive literature search was conducted across PubMed and Scopus electronic databases to identify relevant articles published up to 2024. Inclusion criteria encompassed studies comparing motivational interventions targeting oral hygiene behaviours, with a focus on periodontal clinical indices. Twelve articles meeting the eligibility criteria were selected for analysis. Quality assessment and data extraction were performed systematically.

**Results:** The synthesis of findings from the selected studies revealed a consistent positive effect of motivational interventions on periodontal clinical indices. These interventions encompassed various strategies, including educational sessions, personalized feedback and motivational interviewing. Improvement in indices such as plaque index (PI), gingival index (GI) and periodontal probing depth was observed following motivational interventions, despite the variety of motivational protocols employed.

**Conclusion:** Motivational interventions are effective in enhancing oral hygiene practices and improving periodontal clinical indices. Tailored motivational approaches can serve as valuable tools in promoting oral health behaviours among individuals, potentially reducing the risk of periodontal diseases. Further research is warranted to explore the long-term sustainability and scalability of motivational interventions in diverse populations and settings.

## 1. Introduction

Behavioural change is a key point determining the success or failure of dental treatment plans involving a therapeutic alliance between patients and dental professionals [1].

The choice of appropriate goals with the patient, along with active patient engagement in behavioural change, initiates the journey towards lifestyle change under the guidance and coaching of oral health professionals [2].

Indeed, oral health encompasses various dimensions, such as communication, facial expressions, sensory experiences and functional activity, and is subject to change based on individual experiences, perceptions, expectations and adaptability to different situations [3]. Oral health perception is shaped by personal and communal values and attitudes, reflecting physiological, social and psychological factors crucial for overall well-being [4].

From this perspective, communication becomes fundamental as it involves various aspects of the relationship with the patient, and various health models can be applied in oral health behaviour modification [5].

Concordance, compliance and adherence are terms often used in the context of healthcare to describe patients' behaviour towards prescribed treatments or medical advice. While they share similarities, they also have nuanced differences in meaning.

Concordance refers to a collaborative approach between healthcare professionals and patients in making decisions about treatment. It emphasises mutual understanding, agreement and cooperation between the patient and the healthcare provider regarding the treatment plan. Concordance recognises the importance of patient autonomy and involves a partnership where both parties work together to achieve the best possible outcomes [6].

Compliance describes the extent to which patients follow the recommendations provided by healthcare professionals regarding treatment, medication, lifestyle changes or other aspects of their healthcare regimen. It typically implies a more passive role for the patient, where they adhere to the prescribed regimen without necessarily being fully engaged in the decision-making process [7, 8].

Adherence refers to the extent to which patients follow the recommended treatment regimen, including taking medications as prescribed, following dietary or lifestyle recommendations, attending appointments and engaging in other aspects of their healthcare plan. Adherence encompasses both the behavioural aspect of following the prescribed regimen and the attitudinal aspect of commitment to treatment goals [9].

Proper communication, as well as an increase in the time spent engaging a conversation with patients, has been proved to significantly contribute to an overall improvement in oral health through behavioural change [10–12].

In this scenario, the dental hygienist should take on the role of a health and wellness coach, which entails assisting patients in activating their internal strengths and external resources to bring about behavioural changes aimed at a healthy and sustainable lifestyle through a patient-centred approach. Patients are thus involved in defining their own goals, participate in self-discovery processes or active learning and autonomously monitor their behaviours to increase accountability.

However, healthcare professionals often overlook patients' desire for information, leading patients to report dissatisfaction with their conversations with experts due to the inability to address topics they consider important during visits [13]. Indeed, experts often interrupt patients' narratives after only a few seconds, with 75% of arguments being lists of symptoms within this brief timeframe [14]. A good relationship with the patient is established when communication is symmetrical and alternates between both parties [15]. Vogel, Meyer and Harendza [16] have observed how a professional's communication style is crucial for retaining patients, as it builds the initial impression by focusing on their communication style.

The aim of the present systematic review was therefore to evaluate the effects of oral hygiene motivational interventions in terms of periodontal health outcomes.

## 2. Materials and Methods

### 2.1. Study Protocol

The research was following the Cochrane Handbook and reported according to the PRISMA guidelines [17–19].

The focused question was phrased as it follows:

‘Can concordance between professional and patient lead to better oral health (primary outcome) or are other strategies such as motivation, compliance and adherence more effective?'

The PICOS was devised as it follows:

– (P): Patients enrolled in dental settings.
– (I): Application of motivational and relational techniques.
– (C): Comparison with a control group.
– (O): Evaluation of clinical indices.
– (S): Case–control studies, cross-sectional studies, cohort studies and clinical trials.

Studies published between 2000 and 2024 were included. Only articles in English were included. Studies were excluded if (I) the effect of patient choice in the treatment plan was not reported; (II) the effect of motivation and patient compliance/adherence were not reported and (III) describing in vitro or animal studies.

### 2.2. Literature Search

An electronic search was conducted by three independent calibrated reviewers (Cohen's *κ* > 0.8) on PubMed and Scopus using combinations of controlled terms (MeSH) and free text words. The search strategy was designed as it follows:

“concordance” AND “dentistry”; “concordance” AND “dental care”; “concordance” AND “oral health”; “concordance” AND “oral hygiene”; “concordance” AND “oral care”; “concordance” AND “toothbrush”; “concordance” AND “interdental brushes”; “patient choice” AND “dentistry”; “patient choice” AND “dental care”; “patient choice” AND “oral health”; “patient choice” AND “oral hygiene”; “patient choice” AND “oral care”; “patient choice” AND “toothbrush”; “patient choice” AND “interdental brushes”; “motivation” AND “dentistry”; “motivation” AND “dental care”; “motivation” AND “oral health”; “motivation” AND “oral hygiene”; “motivation” AND “toothbrush”; “motivation” AND “interdental brushes”; “motivation” AND “oral care”; “adherence” AND “dentistry”; “adherence” AND “dental care”; “adherence” AND “oral health”; “adherence” AND “oral hygiene”; “adherence” AND “toothbrush”; “adherence” AND “interdental brushes”; “adherence” AND “oral care”; “compliance” AND “dentistry”; “compliance” AND “dental care”; “compliance” AND “oral health”; “compliance” AND “oral hygiene”; “compliance” AND “toothbrush”; “compliance” AND “interdental brushes”; “compliance” AND “oral care”.

### 2.3. Literature Analysis

Title and abstract analysis of potentially eligible studies was performed by three independent calibrated reviewers (Cohen's *κ* > 0.8). Any disagreement was resolved by discussion. Cohen's *K*-score was calculated to assess the inter-reviewer reliability in the screening phase. Relevant articles meeting the inclusion criteria underwent full text analysis. Following full text analysis, the data were collected and synthesised in evidence tables.

## 3. Results

### 3.1. Study Selection

**Figure 1:**
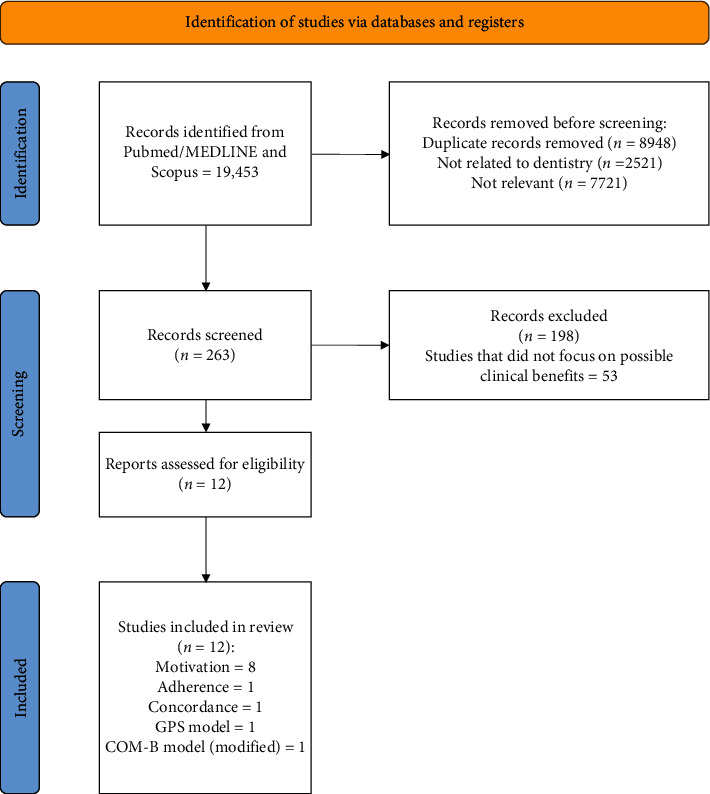
PRISMA flowchart.

The literature search retrieved 19,453 titles. After the removal of duplicates, 8948 items were available. Following title and abstract analysis, 263 articles were deemed eligible for full text analysis, which lead to the final inclusion of 12 articles [20–31] (Figure 1).

### 3.2. Study Population

The included studies were published between 2009 and 2022. Study population was composed by 763 adult patients (mean age: 43.25 years, SD: 9.63). In three studies [19, 20, 28], 210 paediatric patients were enrolled (mean age: 12.08 years, SD: 1.85). Two studies involved the usage of toothbrush [20, 21], three studies [22–24] assessed a combination of toothbrushing and interdental brushing and seven studies [25–31] focused on a motivational approach regardless of the type of hygiene measures employed (Table 1).

### 3.3. Motivational Interventions and Toothbrushing

In two studies [20, 21], 120 patients performing oral hygiene with electric or manual toothbrushes were analysed and the effects of additional motivational and relational techniques were assessed. Plaque index (PI) was found to improve in both studies. Gingival index (GI) was reported in one study [21] and was reduced at follow-up. Although reinforcement of oral hygiene instructions was effective in reducing PI and GI, the limited number of studies hindered the definition of the most effective motivational technique.

### 3.4. Motivational Interventions and Toothbrushing + Interdental Brushing

Three studies [22–24] assessed the effect of cognitive behavioural principles and motivational interviewing in 313 patients performing oral hygiene with toothbrush and interdental brushes. Two studies [22, 23] reported data on the same cohort of patients.

The studies reported variations in PI [22–24], GI [22], probing pocket depth [23] and bleeding on probing [22, 24]. In all the studies, clinical indices were reported to improve when reinforcement was performed.

### 3.5. Motivational Interventions and Clinical Indices

The remaining seven studies [25–31] focused on the motivational approach employed in 540 patients. Fjellström, Yakob and Soder [25] applied cognitive behavioural therapy and found increased adherence to oral hygiene and knowledge about gingivitis. Stenman et al. [26] found no additional effects of a single session of motivational interviewing on clinical indices, and the same outcome was reported by Brand et al. [27]. Conversely, Oruba et al. [28] described an improvement in clinical parameters in patients with higher self-reported motivation. Alijaaba, McDonald and Newton [29] reported an improvement in clinical parameters in orthodontic patients and enhanced adherence to clinician's recommendations. Asimakopoulou et al. [30] behavioural intervention using individualised periodontal disease risk communication, with or without GPS, reduced plaque and bleeding and increased interdental cleaning. Finally, Gunpinar and Melaci [31] assessed the effect of a periodontal health education session versus standard oral hygiene education. The authors found that motivational interventions lead to an improvement in oral hygiene.

## 4. Discussion

### 4.1. Main Findings

The present review highlights an overall beneficial effect of motivational interventions in the improvement of oral hygiene and clinical indices of plaque accumulation and inflammation. However, it should be noted that the quality of evidence is low due to the heterogeneity of motivational approaches and protocols employed. Importantly, current literature highlights that only the minority of studies actually refers to concordance; thus, this study highlights the unmet need for structured motivational intervention to improve oral health outcomes.

The effectiveness of motivational interventions in improving oral hygiene and periodontal outcomes varies depending on the frequency, timing and the method of delivery.

Toothbrush and dental floss or interproximal brushes motivation can improve oral health, but the motivation must be repeated to be efficient [32–35]. Studies, such as those by Marini et al. [20] and Jönsson et al. [22, 23], demonstrate that repeated oral hygiene instructions and motivation are crucial in reducing PI and improving periodontal health. Continuous reinforcement of these motivational messages, rather than a single session, leads to better long-term adherence to oral hygiene practices, particularly in patients undergoing orthodontic treatment or managing chronic periodontitis. This suggests that motivation should be an ongoing process throughout the treatment, rather than a one-time intervention.

Additionally, the timing of these motivational sessions is important; for instance, Gunpinar et al. [31] found that educational sessions before the onset of severe periodontal disease can significantly improve patient outcomes. However, single and stand-alone sessions, as shown in the study by Stenman et al. [26], might not be sufficient for sustained behavioural change. While the timing of effective patient motivation is still to be defined due to the heterogeneity of studies in the literature, it appears that in the short-term motivation might be useful in the promotion of dental and periodontal health [20, 21, 27, 28].

The delivery of these interventions is also crucial—cognitive behavioural approaches and motivational interviewing techniques, when conducted by trained professionals, such as dental hygienists or periodontists, have shown superior effectiveness in promoting oral health behaviours compared to traditional methods. Although motivational interviewing seems to influence periodontal parameters, it strictly depends on the professionalism of the healthcare provider and the empathy established in the dental professional–patient relationship. Collaboration, evocation and autonomy must be the main focuses of the interview so that the patient may make his own decisions and understand that he is the primary creator of the change in his health [36, 37].

The studies collectively suggest that for optimal periodontal health outcomes, motivation should be frequent, well-timed and delivered by professionals using evidence-based psychological techniques.

### 4.2. The Role of Motivation

Different perspectives on motivational techniques, timing of motivation sessions and the analysis of psychological traits have been previously reported in the literature.

Although several models have been discussed in dentistry [38–40], limited correlation with periodontal health (PI, bleeding and probing depth) has been provided to date. Interventions that target patient beliefs, motivation, intention and self-regulation may, within the bounds of the evidence now available, improve periodontal health outcomes. However, the variations in protocol design, research population and follow-up periods hinder the direct comparison between different motivational therapeutic approaches [39].

Indeed, an increase in patient motivation leads to an increase in compliance levels [41], and despite the lack of consistency among studies in terms of type and frequency of instruction and motivation, higher compliance encourages the improvement in clinical outcomes [42–45].

Planned behaviour can affect the psychological variables of the patient and favour adherence to oral hygiene instructions, being able to improve oral health [46–48].

Tracking clinical indices over time increases the involvement and cooperation of the patient in the treatment. Therefore, therapeutic alliance is essential to the effectiveness of periodontal therapy. Without the continuous support and communication provided by dental professionals to the patient, the therapy will not be effective over the short and long term [36, 49, 50, 51].

Goal setting, planning and self-monitoring are successful methods in promoting behavioural change in oral health, especially in patients with periodontitis. This approach is also beneficial in the long run as it encourages adherence to oral hygiene practices, particularly regarding interproximal surfaces and the reduction of plaque and bleeding scores [52–54].

Another strategy to promote the oral health of patients is adherence to behavioural instructions on oral hygiene, which allows to reduce periodontal indices [55, 56].

In the literature, there is scarce evidence on the effect of concordance on the clinical benefits of the patient, as patients' involvement in the choice of treatment improves adherence to the treatment and favours the understanding of the patients' expectations and desires [57].

### 4.3. Motivation in Dentistry

In dentistry, it is essential for the patient to be motivated to adopt behaviours that responsibly support oral health [58]. These include adhering to healthy lifestyles and habits (having a balanced diet, engaging in physical activity, avoiding tobacco and alcohol abuse), regularly attending check-ups and oral hygiene sessions, adhering to proposed treatment plans and maintaining good plaque control through home oral hygiene [59]. Indeed, no type of dental treatment, nor oral health itself, can be maintained over time without active collaboration in their upkeep [60].

In addition to being intrinsically motivated, the patient must also be encouraged and supported by the attending clinician; therefore, motivating means establishing a relational process facilitating patient awareness of personal needs and experiences [61]. Dental professionals should assist patients in activating their internal strengths and external resources to make behavioural changes aimed at a healthy and sustainable lifestyle through a patient-centred approach, where patients are involved in defining their own goals, participate in self-discovery or active learning processes and autonomously monitor their behaviours to increase accountability. Coaching is thus conceived to provide social nourishment, supporting autonomy, developing skills and creating a trusting partnership. This approach helps stimulate the natural human tendency towards interest, growth and self-control.

To date, there is no superior motivation model for effectively conducting motivational interventions. This may be due to clinicians' limited ability to apply psychological theories of motivation in the dental context and the need for more methodologically rigorous studies examining not only their impact on clinical indices but also self-reported measures of individual behaviours [62].

Therefore, valid models exist capable of acting on short-term clinical indices and improving periodontal health conditions, especially in the presence of chronic conditions like periodontitis, but they are still unclear and no one model is better than the others in long-term maintenance.

### 4.4. Strategies for Behavioural Changes

To reduce the communicative gap between dental professionals and patients, it is necessary to create a welcoming listening space where the patient can express doubts, fears, beliefs and even habits [63]. While information for improving the patient's oral health must be delivered professionally, the language should also be suitable for the recipient. In conveying openness, a calm and reassuring tone of voice should be used to create a comfortable environment, instil confidence in the patient, convey serenity and security, promote more effective communication, make the patient more likely to listen and feel at ease [64]. Personalising treatment should not only focus on the patient's clinical needs but also on the type of approach the patient needs, ensuring that preventive therapies are welcomed enthusiastically and not perceived as coercion. Even oral hygiene measures should be chosen, considering the patient's perception of them and taking care not to propose too many goals to achieve simultaneously initially [65]. The pursuit of the protocol, which should be simple, feasible and practicable, should be made sustainable mentally, physically and economically for the patient, in the light of a tailored approach to treatment [66].

Motivation involves the achievement of a positive behavioural change through individual responsibility, environmental control, personal gratification and social support. It is essential to note that behavioural change is an individual and dynamic process. There is no universal formula and success depends on personal variables, including motivation, social support, available resources and the ability to face challenges with self-efficacy [67]. Self-efficacy affects perceived behaviour of the patient as the greater the awareness of being able to carry out a long-term action, the more easily that behaviour will be implemented [59].

### 4.5. Strategies to Improve Motivation

Once the patient's motivational level regarding their improvement has been understood, targeted learning strategies can be used to meet the specific needs of that patient.

It is important to choose and personalise the treatment goals based on the specific needs of the patient and adapt them during the programme based on the progress and challenges encountered [68]. Clarity of goals will contribute to the success of the programme in promoting positive behavioural change.

The ability to personalise treatment goals is essential, as it allows the practitioner to cater to the unique physiological and pathological variables of each patient, such as age, gender and overall health status. Customisable and modifiable protocols must be tailored to the patient's specific oral health conditions and adjusted over time to reflect changes in their health status. This approach necessitates ongoing monitoring and regular motivational reinforcement to maintain behavioural changes that support long-term oral health. The involvement of other healthcare and nonhealthcare professionals is also crucial, as a multidisciplinary approach can provide comprehensive support to the patient, ensuring that all aspects of their health are considered in the treatment plan [68] (Table A1).

**Figure 2:**
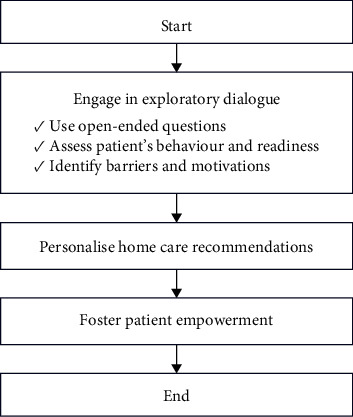
Communication scheme to improve motivation.

Empowerment of the patient is a key outcome of effective communication, enabling them to take control of their oral health. By fostering internal empowerment, where the patient gains awareness and control over their health choices, the dental hygienist helps to build the patient's confidence and responsibility in maintaining their oral health. This holistic approach, combining ethical knowledge with strong communication skills, is essential for achieving optimal outcomes in dental hygiene practice [69] (Figure 2).

### 4.6. Study Limitations

The present study has some limitations. One significant challenge was the limited amount of available literature, which, coupled with the diversity in motivational methods employed across the included studies, restricted our ability to conduct a comprehensive comparison. Additionally, there was considerable variability in the outcomes reported, further complicating efforts to draw consistent conclusions. Specifically, data related to plaque accumulation and gingival inflammation were recorded using different methods across studies, which prevented us from performing a meta-analysis. Furthermore, the varying timepoints for recall sessions across the studies introduced another layer of complexity, making direct comparisons between studies particularly challenging. These factors collectively limited the overall robustness and generalizability of our findings.

## 5. Conclusions

The data presented suggest that effective motivational interventions are essential for improving oral hygiene and periodontal outcomes. The success of these interventions relies on their frequency, with repeated and continuous reinforcement proving more effective than single or sporadic sessions. Additionally, the timing of these interventions, particularly when introduced before the onset of severe periodontal issues, enhances their impact. The delivery method also plays a crucial role, with motivational techniques based on cognitive behavioural principles and conducted by trained professionals yielding the most significant improvements. Overall, to achieve optimal periodontal health, motivation should be integrated as a consistent, well-timed component of oral healthcare, tailored to individual needs and administered by skilled practitioners. As in the literature, there is still limited mentioning of concordance in oral hygiene; further research is needed to identify the most suitable motivational technique to increase the levels of concordance in the performance of oral hygiene.

## Figures and Tables

**Table 1:** Synthesis of the included studies.

Authors	Year	Study design	Sample	Follow-up (months)	Aim	Study groups	Outcomes	Clinical outcomes
Marini et al. [20]	2014	RCT	60	5	Effects on PI scores of manual or electric toothbrush with or without repeated OHI and motivation on patients wearing fixed orthodontic appliances	Group A: electric toothbrush + OHI (all times) Group B: electric toothbrush + OHI (at baseline) Group C: manual toothbrush + OHI (all times) Group D: manual toothbrush + OHI (at baseline)	Repeated OHI and motivation are crucial in reducing PI score in orthodontic patients, independent of the type of toothbrush used	PI scores with electric of manual toothbrushes obtained with the use of OHI (all times) were similar, with a lower reduction

Yıldırım and Kayaaltı-Yüksek [21]	2020	RCT	60	0.5	Effects of different motivational methods on children's oral hygiene and periodontal health	Group A: toothbrushing + music video (A1 electric, A2 manual) Group B: toothbrushing + hourglass timer (B1 electric, B2 manual) Group C: control (OHI; C1 electric, C2 manual)	Statistical differences between groups weren't found. A significant decrease in clinical indices was found in all groups.	PI decreased in all groups, but the highest reduction was obtained in video-group (both electric and manual toothbrush). In video-group, the reduction was 51.8% for manual toothbrush and 55% for electric toothbrush

Jönsson et al. [22]	2009	RCT	113	12	Effectiveness of ITOHEP for oral hygiene (electric toothbrush and dental floss and triangular wood sticks) in patients with chronic periodontitis compared with standard treatment	Group A: ITOHEP based on cognitive behavioural principles and motivational interviewing Group B: standard OHI	The ITOHEP was efficacious in improving long-term adherence to oral hygiene in periodontal treatment	Plaque and gingival status were improved at the end of the follow-up in both groups. Plaque scores had a gain of 78% in test group, and 71% for global GI

Jönsson et al. [23]	2010	RCT	113	12	Effectiveness of ITOHEP for oral hygiene (electric toothbrush and dental floss and triangular wood sticks) on periodontal health compared with standard OHI	Group A: ITOHEP based on cognitive behavioural principles and motivational interviewing Group B: standard OHI	ITOHEP intervention in combination with scaling is preferable to the ST programme in non-surgical periodontal treatment.	Plaque score, probing pocket depth and bleeding on probing were reduced, without differences between groups. Probing pocket depth reduction, at 3 months, was 12% in ITOHEP and 14% in ST group

Nardi et al. [24]	2016	RCT	200	1	Effectiveness of TBM (manual toothbrush and interdental brushes)	Group A: TBM Group B: standard protocol	TBM (based on concordance between professionals and patients) improves clinical indices	TBM reduced PI and BoP

Fiellstrom, Yakob and Soder [25]	2010	Pilot study	4	0.5	Increase the adherence to oral hygiene instruction	Group A: CBT Group B: traditional instruction	Using a modified model of CBT, resulted in increased adherence to oral hygiene and knowledge about gingivitis, compared with traditional instructions	GI had improved from 2 to 0 in test group, with a higher decrease of PI

Stenman et al. [26]	2012	RCT	44	6	Evaluate the effect of a single session of MI on self-performed periodontal infection control	Group A: single session of MI before non-surgical periodontal therapy Group B: conventional educational intervention before non-surgical periodontal therapy	A single freestanding MI session as a prelude to conventional periodontal treatment had no significant effect on the individuals' standard of self-performed periodontal infection control in a short-term perspective	At the end of follow-up, after session of information and OHI, MBI reduction was 19% (18% in control group) and PI reduction was 25% (19% in control group)

Brand et al. [27]	2013	RCT	56	3	Evaluate whether the use of BMI is effective in improving internal motivation for oral hygiene behaviour	Group A: BMI + TOHE Group B: TOHE alone	Statistically differences between groups weren't found. A single session of motivational interviewing is sufficient	BoP decreased by 19% in TOHE group and by 17% in BMI; similar results were found for PI and the change of periodontal pockets

Oruba et al. [28]	2014	Cross-sectional	199	NR	Examine whether the motivation of patients suffering from chronic periodontitis influences their clinical periodontal condition	Questionnaire concerning patients' medical and dental history, modified Zychlińscy motivation assessment questionnaire, clinical periodontal examination	Periodontal patients with greater motivation having better oral health	Patients with highest API index (>70%) had significantly lower motivation scores; this is similar also for BoP

Aljabaa, McDonald, and Newton [29]	2016	RCT	90	4.5	Enhance adherence to oral hygiene instructions in orthodontic patients	Group A: treatment ad usual (TAU) Group B: treatment ad usual (TAU) + mind mapping Group C: treatment ad usual (TAU) + if-then planning	All 3 techniques enhanced adherence, but no single method proved superior to any other	There was a change in plaque levels during follow-up, unlike BoP

Asimakopoulou et al. [30]	2019	RCT	97	3	Determines the effects of a routine TAU versus a risk communication intervention (Risk) versus a GPS intervention on periodontal disease patients' clinical and psychological outcomes	Group A: Periodontal disease risk using only the Previser Risk Calculator Group B: periodontal disease risk using only the Previser Risk Calculator + GPS-behavioural intervention	A simple behavioural intervention using individualised periodontal disease risk communication, with or without GPS, reduced plaque and bleeding and increased interdental cleaning over 12 weeks	The GPS group showed a similar pattern of plaque reduction to the risk group with significant reduction at 4 weeks (from 16.23% to 10.91%), and again at 12 weeks (to 9.65%). BoP and probing pocket depth also decreased

Gunpinar and Meraci [31]	2022	RCT	50	6	Evaluate the effect of a PHES, which included education on the pathogenesis and consequences of periodontal diseases on oral hygiene motivation in patients with gingivitis	Group A: periodontal health education session Group B: standard oral hygiene education	Increased knowledge and awareness about periodontal diseases and its consequences, including systemic effects, via educational MI session increased intrinsic motivation and improved oral hygiene of patients with gingivitis, especially regarding interproximal surfaces	Regarding RMNPI, there was a reduction of 0.50 in group with periodontal health education session and a reduction of 0.42 in group with standard oral hygiene education

Abbreviations: BMI, brief motivational interviewing; BoP, bleeding on probing; CBT, cognitive behavioural therapies; GI, gingival index; GPS, goal-setting, planning and self-monitoring; ITOHEP, individually tailored oral health educational program; MBI, marginal gingival bleeding index; MI, motivational interviewing; NR, not reported; OHI, oral hygiene instructions; PHES, periodontal health education session; PI, plaque index; RCT, randomized clinical trial; RMNPI, rustogi modified navy plaque index; TAU, treatment as usual; TBM, tailored brushing method; TOHE, tailored oral health education.

**Table 2:** Skills of the dental professional to implement treatment strategies.

Treatment strategies	
Knowledge	

Professional ethics	• Necessary to establish long-term trust relationships • Privacy of sensitive data • Absence of prejudice

Medical evidence and its continuous updating	• Therapeutic appropriateness in terms of effectiveness, efficiency and safety, free from any interest or conditioning from the operator • Guarantee of patient's fundamental rights • Knowledge of evidence to personalise therapies appropriately according to physiological and/or pathological variables, signs, symptoms and systemic correlations that can vary based on age, gender, race and related health status

Oral care technologies and devices	• Constant update on technological progress • Principle of maximum achievable performance with minimal effort • Cost-effectiveness

Application of customisable and modifiable protocols during therapy	• Choice of home therapy and useful tools based on the physiological and/or pathological variables • Assessment of manual skills and character inclinations that may condition the acceptance of devices • Personalisation of home therapy depending on age and systemic conditions and periodical monitoring • Motivational reinforcement recall to support and encourage patients towards improvements and progress

Involvement of other healthcare and nonhealthcare professionals who may be involved in the patient's health process	• Motivational dialogue with the involvement other figures in the patient's health sphere, such as caregivers, parents if the patient is a minor, guardians, the primary care physician, the referring pharmacist, the gynaecologist if the patient is pregnant, the nutritionist, et cetera • The entire dental team must show the same interest in the patient

Principles of effectiveness, efficiency and safety	• Setting therapeutic goal • Efficiency with the fewest possible devices • Safety guaranteed through devices certifications (e.g., ISO and CE) • Ergonomics adequate to patient specificity

Communication skills	

Empathic ability	• Empathy to tune the operator and the patient into a common language • Emotional and empathetic participation

Capacity for active listening	• Verbal capacity • Active and participatory listening

Verbal and nonverbal communication skills	• Verbal communication through open, sincere, honest and clear dialogue • Nonverbal communication considering the age, gender, patient's potential and current health conditions • The context in which communication takes place should be welcoming, not noisy, with soft lighting and if not disturbing, with a relaxing background music.

Ability to stimulate empowerment in others	• Focus on empowerment as a set of actions and interventions aimed at strengthening the patient's power of choice through knowledge improvement • Internal empowerment through awareness and control of their choices

## Data Availability

Data sharing is not applicable to this study, as no new data were generated in this study.
